# Plastic pollution of the world’s seas and oceans as a contemporary challenge in ocean governance

**DOI:** 10.1038/s41467-018-03104-3

**Published:** 2018-02-14

**Authors:** Marcus Haward

**Affiliations:** Institute for Marine and Antarctic Studies, University of Tasmania, Hobart, TAS 7005 Australia

## Abstract

The pervasive nature of marine plastic pollution was highlighted at the recent United Nations Environment Assembly. This meeting saw strong commitments for action, but at the same time reinforced the challenges for contemporary ocean governance in addressing marine plastic pollution.

The problem of plastic pollution in the world’s seas and oceans has attracted increasing scientific concern^1^, with calls for an international agreement to address this issue. Any such agreement would extend, complement, and also challenge existing international, regional, national, sub-national, and local initiatives. Responses to the problem of marine plastic pollution will need to involve and link state and non-state actors, business, and civil society, looking to integrated solutions that move away from traditional state-based, sector-focused responses to oceans' issues.

The United Nations Environment Assembly meeting in Nairobi, Kenya, convened in early December 2017 under the auspices of the United Nations Environment Program, was the most recent gathering to address the significant issue of plastic pollution in the world’s seas and oceans. The topic was included in one of the 11 resolutions discussed at the meeting, which, although non-binding, are likely catalysts for further action. For the first time, the United Nations Environment Assembly deliberations included the adoption of a Ministerial Declaration by consensus. This declaration noted that annually “we dump [from] 4.8 to 12.7 million tonnes of plastic in oceans”. The president of the assembly noted that “the results … will provide us with concrete solutions to achieve our aspirations”^2^.

## Marine plastic pollution

Although there is broad-based recognition of the problem of marine pollution, the challenge in addressing marine plastic pollution reflects the complexity of a multi-faceted problem. A vast majority of marine plastic pollution derives from land-based sources (4.8–12.7 million metric tonnes of plastic annually)^3^, so a sole focus on marine oriented solutions is insufficient. The sources of such plastics are equally diverse, from commercial and recreational ships and vessels, fishing and aquaculture operations (rope, waste, fishing gear, nets) to land based sources, street litter, dumping, packaging (including plastic bags), plastic sheeting and production waste^3^.

Our growing understanding of the insidious and deleterious impacts of micro-sized (1 µm–1 mm) and nano-sized (<1 µm) plastic particles has further emphasised the environmental threat marine plastics pose. Microplastics are derived either from small particles developed for specific applications, or produced through the breakdown of larger items^4^. Micro-sized and nano-sized plastic particles are increasingly being consumed by marine life that confuse them with food sources^1^. These particles specifically, and plastic pollution in general, are being found in marine life in isolated areas indicating the pervasive nature of such pollution.

## Issues for contemporary ocean governance

Marine pollution has long been recognised as a threat and a catalyst for ongoing developments in ocean governance. One major means to address and combat these threats has been through the work of international bodies such as the International Maritime Organization (IMO). The IMO, responsible for the administration of the International Convention for the Prevention of Pollution from Ships 1973/78 (MARPOL), the Convention on the Prevention of Marine Pollution by Dumping of Wastes and Other Matter 1972 and its 1996 Protocol (London Convention/Protocol), have also recognised the problem of plastic pollution and marine litter.

Ship-sourced disposal of plastics is prohibited under the MARPOL convention for vessels flagged to parties to this convention in both exclusive economic zones and waters beyond national jurisdiction. The enforcement of MARPOL provisions needs strong monitoring, control and surveillance systems to ensure effective compliance, a key role for the flag state of vessels, from the smallest fishing vessel to large supertankers. Land-based sources of pollution entering the marine environment, which are by far the main source of marine plastic pollution, need similar monitoring and control by states.

Given the character of the problem, attention has focused on international action with measurable targets to reduce macro-plastic and micro-plastic marine pollution^1^. The global community has risen to similar challenges in the past. Fifty years ago, Arvid Pardo, Malta’s Ambassador to the United Nations, called for concerted actions to address what he saw as the potential for uncontrolled exploitation of the world’s oceans, threatening what Pardo recognised as areas of for the ‘common heritage of mankind’. Pardo’s calls for action led to changes in ocean governance, most notably the Third United Nations Conference on the Law of the Sea that concluded with the drafting and eventual entry into force of the United Nations Law of the Sea Convention.

An international agreement to address marine plastics could be pursued in a similar manner, but necessarily in a more integrated and broad-based approach than that attempted in the late 1960s. It could be modelled on the successful Montreal Protocol addressing ozone-depleting substances that saw replacement of chlorofluorocarbons and an increasing public awareness of the problem. A key starting point is to build on commitments made at Nairobi, reaffirming the principles contained in the Rio Declaration on Environment and Development in 1992^4^, and the more recent commitments made in 2015 by world leaders in adopting the 2030 Agenda for Sustainable Development which, inter alia, includes 17 Sustainable Development Goals (SDGs)^5^. SDG 14, ‘Conserve and sustainably use the oceans, seas and marine resources for sustainable development’, provides a focus for ongoing action on marine plastic pollution.

Another related direction (advanced by work at the United Nations Environment Assembly) is to build on the commitments made at the United Nations Conference to Support the Implementations of SDG 14, also known as the Oceans Conference, held in New York in June 2017. One outcome of the Oceans Conference was the creation of Communities of Ocean Action, with marine pollution identified in SDG Target 14.1 being the clear focus of such work. A side event at the United Nations Environment Assembly was a forum for governments, non-governmental organisations and civil society groups to present innovative solutions to tackle marine pollution through voluntary commitments by members of Communities of Action.

Scientific support can continue to be encouraged by organisations such as the Joint Group of Experts on the Scientific Aspects of Marine Environmental Protection (GESAMP). Ongoing scientific work must continue in order to understand the scale and scope of the problem. However, adopting a precautionary approach would mean that this uncertainty should not be used to limit immediate efforts to reduce impacts and to mitigate their effects. Linking business and industry to such issues could be facilitated by the work of the World Ocean Council as a global industry alliance committed to Corporate Ocean Responsibility (www.oceancouncil.org).

Community action can include initiatives that reduce the amount of plastic entering the marine environment, a focus on recycling and reusing plastics, and continue to improve public awareness of the impacts and vectors of marine plastic pollution, as well as practical mechanisms such as litter traps. These initiatives are independent of, but complement more globally focused actions at the state level^6^. Civil society action links to market and economic drivers that see consumer demand force a reduction in level and type of plastic. Industry can be encouraged to develop and support codes of conduct and certification of practices, along the lines of successful standard setting and certification developing in a variety of other sectors. Such certification supports and reinforces industry corporate social responsibility and social license. This in turn links back to appropriate and effective government regulation, particularly addressing the management of single use plastics and micro-sized and nano-sized plastic particles.

## A way forward

There remains a number of challenges in addressing the problem of marine plastic pollution. In 1967, calls for a refocus on the ‘common heritage’ of the world’s seas and oceans led to concerted and revolutionary action by the world community to address concerns and challenges. In late 2017, the United Nations Environment Assembly resolution on marine plastic pollution serves a similar purpose. The meeting, with broad based agreement from participating states and non-governmental organisations, may well provide the impetus for ongoing action to combat marine plastic pollution.

International agreements are not easily developed and are often criticised for the time taken to reach agreements and the tendency for a minimal tolerable consensus to shape outcomes. Despite these criticisms, the international framework for ocean governance continues to evolve. International initiatives addressing marine plastic pollution need to be supported by strong and focused scientific research, the engagement of business and community organisations, as well as engaged and committed government action on different scales, supporting community-based programs that address the use and disposal of plastics.
